# The respiratory oxygenation index for identifying the risk of orotracheal intubation in COVID-19 patients receiving high-flow nasal cannula oxygen

**DOI:** 10.62675/2965-2774.20240203-en

**Published:** 2024-06-14

**Authors:** Aline Braz Pereira, Felipe Dal Pizzol, Viviane Cordeiro Veiga, Leandro Utino Taniguchi, Aline Finoti Misquita, Gustavo Augusto Couto Carvalho, Ligia Maria Coscrato Junqueira Silva, Michelli Marcela Dadam, Ruthy Perotto Fernandes, Israel Silva Maia, Cassio Luis Zandonai, Alexandre Biasi Cavalcanti, Marcelo Luz Pereira Romano, Glauco Adrieno Westphal

**Affiliations:** 1 Centro Hospitalar Unimed Intensive Care Unit Joinville SC Brazil Intensive Care Unit, Centro Hospitalar Unimed - Joinville (SC), Brazil.; 2 Universidade do Extremo Sul Catarinense Postgraduate Program in Health Sciences Criciúma SC Brazil Postgraduate Program in Health Sciences, Universidade do Extremo Sul Catarinense - Criciúma (SC), Brazil.; 3 BP - A Beneficência Portuguesa de São Paulo Intensive Care Unit São Paulo SP Brazil Intensive Care Unit, BP - A Beneficência Portuguesa de São Paulo - São Paulo (SP), Brazil.; 4 Universidade de São Paulo Hospital das Clínicas Faculdade de Medicina São Paulo SP Brazil Intensive Care Unit, Hospital das Clínicas, Faculdade de Medicina, Universidade de São Paulo - São Paulo (SP), Brazil.; 5 Hospital Municipal São José Intensive Care Unit Joinville SC Brazil Intensive Care Unit, Hospital Municipal São José - Joinville (SC), Brazil.; 6 Hospital Nereu Ramos Intensive Care Unit Florianópolis SC Brazil Intensive Care Unit, Hospital Nereu Ramos - Florianópolis, Santa Catarina (SC), Brazil.; 7 Hcor-Hospital do Coração Intensive Care Unit São Paulo SP Brazil Intensive Care Unit, Hcor-Hospital do Coração, Associação Beneficente Síria - São Paulo (SP), Brazil.

**Keywords:** COVID-19, Coronavirus infections, Cannula, Intubation, Respiratory insufficiency, Respiratory rate, Oxygen

## Abstract

**Objective:**

To assess whether the respiratory oxygenation index (ROX index) measured after the start of high-flow nasal cannula oxygen therapy can help identify the need for intubation in patients with acute respiratory failure due to coronavirus disease 2019.

**Methods:**

This retrospective, observational, multicenter study was conducted at the intensive care units of six Brazilian hospitals from March to December 2020. The primary outcome was the need for intubation up to 7 days after starting the high-flow nasal cannula.

**Results:**

A total of 444 patients were included in the study, and 261 (58.7%) were subjected to intubation. An analysis of the area under the receiver operating characteristic curve (AUROC) showed that the ability to discriminate between successful and failed high-flow nasal cannula oxygen therapy within 7 days was greater for the ROX index measured at 24 hours (AUROC 0.80; 95%CI 0.76 - 0.84). The median interval between high-flow nasal cannula initiation and intubation was 24 hours (24 - 72), and the most accurate predictor of intubation obtained before 24 hours was the ROX index measured at 12 hours (AUROC 0.75; 95%CI 0.70 - 0.79). Kaplan-Meier curves revealed a greater probability of intubation within 7 days in patients with a ROX index ≤ 5.54 at 12 hours (hazard ratio 3.07; 95%CI 2.24 - 4.20) and ≤ 5.96 at 24 hours (hazard ratio 5.15; 95%CI 3.65 - 7.27).

**Conclusion:**

The ROX index can aid in the early identification of patients with acute respiratory failure due to COVID-19 who will progress to the failure of high-flow nasal cannula supportive therapy and the need for intubation.

## INTRODUCTION

The high-flow nasal cannula (HFNC) is an oxygen supply system that can deliver up to 100% heated and humidified oxygen through the nasal interface at a maximum flow rate of 60L/minute (some devices allow a maximum flow of 80L/minute).^(1)^ In addition to enabling the supply of high fractions of inspired oxygen (FiO_2_), the use of HFNC can improve ventilatory efficiency, reduce dead space and favor a decrease in carbon dioxide.^(2)^

Oxygen therapy with a HFNC has been gaining attention as a strategy for noninvasive ventilatory support in patients with pneumonia and severe acute hypoxemia. This technique has been associated with improved alveolar ventilation and reduced respiratory effort.^(3-6)^ In patients with coronavirus disease 2019 (COVID-19), HFNC therapy can reduce the need for intubation, as well as the length of intensive care unit (ICU) stay, with no apparent effect on mortality.^(7-9)^ Although the use of HFNC therapy in hypoxemic patients is associated with positive results,^(10,11)^ delayed intubation can lead to poor outcomes, including increased mortality.^(12-14)^ High-flow nasal cannulas have been widely used in and outside the ICU, and recognizing patients who will deteriorate and need ICU admission and mechanical ventilation (MV) is extremely important.^(15)^

The respiratory oxygenation index (ROX index), which is defined as the ratio of peripheral oxygen saturation (SpO_2_) to FiO_2_ divided by the respiratory rate, has been proposed as a measure to identify patients who are at increased risk of failure of noninvasive support with HFNC therapy during hypoxemic respiratory failure. In patients with pneumonia and acute hypoxemic respiratory failure, the ROX index measured within 12 hours after HFNC therapy initiation is a good predictor of a greater risk of HFNC failure.^(16,17)^ Different studies have shown that a ROX index ≤ 4.88 measured in the first hours of HFNC therapy has good discriminatory ability for identifying the risk of intubation in hypoxemic patients,^(18,19)^ including patients with COVID-19.^(20-23)^ The ROX index could reflect a specific moment in time instead of the clinical evolution of the patient, and some authors also suggested that the ROX index score at the time of intubation was associated with improved survival to hospital discharge and may reflect the severity of respiratory disease.^(24)^

However, few studies have analyzed the ROX index in patients with COVID-19. Most of them were single-center studies with small sample sizes. None of the studies were conducted in Brazil. The meta-analysis showed that heterogeneity among studies was high, and different cutoff values of the ROX index were used. The aim of this study was to assess whether the ROX index measured after the start of HFNC therapy can help identify the need for intubation in patients with acute respiratory failure due to COVID-19.

## METHODS

### Study design and setting

This retrospective, observational, multicenter study was conducted at the ICUs of six Brazilian hospitals from March to December 2020. The Research Ethics Committees of all centers approved the study protocol (Ethical Clearance Certificate: 46574321.1.1001.5362). Since this study was retrospective, informed consent was not necessary.

### Participants

We included patients who were older than 18 years, admitted to the ICU, had acute respiratory failure due to confirmed COVID-19, and who were receiving HFNC oxygen therapy. Acute respiratory failure was diagnosed based on the clinical judgment of the teams. The presence of COVID-19 was confirmed by reverse transcription polymerase chain reaction (RT-PCR), antigen testing, or serological testing (IgM positive).^(25,26)^

The exclusion criteria were the presence of acute respiratory failure without laboratory confirmation of COVID-19 and with a more likely alternative diagnosis or laboratory confirmation of another etiological agent; orotracheal intubation on ICU admission; postextubation or postoperative HFNC therapy; end-stage disease or exclusive palliative care; and incomplete records regarding data on the primary outcome.

### Outcomes

The primary outcome was the need for orotracheal intubation within 7 days after the start of HFNC therapy. The secondary outcome was the need for orotracheal intubation within 48 hours after HFNC therapy started. No formal standardization was performed among the participating centers in terms of intubation criteria since no validated criteria for orotracheal intubation of COVID-19 patients were available during the study period.

### Data sources and measurement

The following variables were collected and recorded for analysis: sex, age, and Simplified Acute Physiology Score 3 (SAPS 3). Physiological data such as heart rate, mean arterial pressure, respiratory rate, SpO_2_, FiO_2_, and oxygen flow were recorded at least every two hours on patient monitoring forms at the bedside according to routine procedures of the services. The ROX index was calculated using the following formula: (SpO_2_/FiO_2_)/respiratory rate. The variables were obtained from the medical records, and the ROX index was calculated in the following chronological order: start of HFNC therapy (ROX-baseline) and after 2 (ROX-2 h), 6 (ROX-6 h), 12 (ROX-12 h) and 24 hours (ROX-24 h). Each patient was followed until hospital discharge or death. The data were collected from the patients’ medical records and transferred to a paper or electronic case report form. During and after the end of data collection, research coordinators from the study coordinating center maintained contact with the participating centers to ensure correct completion of the data and the use of reliable information to avoid missing data and to mitigate the risk of bias.

### Sample size

The minimum estimated sample size to confirm or refute the study hypothesis was 243 patients, assuming a type 1 error of 0.05, a type 2 error of 0.20, an area under the receiver operating characteristic curve (AUROC) of 0.80 and an AUROC null hypothesis equal to 0.70, considering an intubation rate of 28-35% based on previous studies.^(18,19,27)^

### Statistical methods

All analyses were performed using MedCalc Statistical Software, version 20.2 (MedCalc Software Ltd., Ostend, Belgium) [https://www.medcalc.org; 2022]. The Kolmogorov-Smirnov test was used to evaluate the distribution of the data. Continuous variables are reported as medians and the respective interquartile ranges (IQRs) and were compared using the Mann-Whitney U test. Categorical variables are presented as absolute and relative frequencies and were compared using the chi-squared test. A p value < 0.05 was considered to indicate statistical significance. However, the results of the secondary outcome and other analyses should be considered exploratory (95% confidence interval [95%CI] and p value) because they were not adjusted for multiple hypothesis testing. We determined the AUROC to determine the ROX index that defines the success or failure of HFNC oxygen therapy at the start of this therapy and after 2, 6, 12 and 24 hours. An AUROC of 0.70 to 0.79 indicates moderate discriminatory ability, and an AUROC ≥ 0.80 indicates excellent discrimination.^(27)^ The respective sensitivity and specificity were also obtained. After defining the best ROX index in the AUROC analysis corresponding to the maximization of Youden's index, Kaplan-Meier curves were constructed to analyze the time to intubation, and the groups were compared using the log-rank test. A sensitivity analysis was performed for the primary outcome among patients who were intubated before and after the median interval between HFNC therapy initiation and intubation to reduce the effect of selection bias. Patients with incomplete records of the primary outcome were excluded from the study.

## RESULTS

### Participants

**Figure 1:**
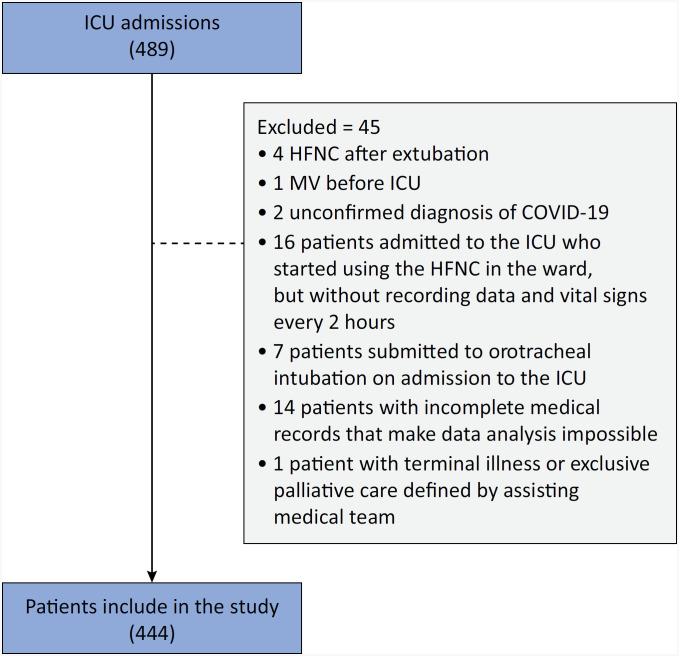
Patients selection.

A total of 489 patients were admitted to ICUs because of respiratory failure due to COVID-19 and underwent HFNC therapy. Of these, 444 met the eligibility criteria and were included in the study (Figure 1); 261 (58.7%) progressed to need intubation. The intubated patients were younger (59 [49 - 73] *versus* 65 [54 - 77] years; p = 0.001), were more critically ill (SAPS 3: 44 [37 - 52] *versus* 42 [36 - 47]; p < 0.001), and more frequently required noninvasive mask ventilation (69.3% *versus* 58.4%; p = 0.01). The groups were similar in terms of comorbidities, Charlson Comorbidity Index (CCI) score, awake prone positioning, and types of oxygen delivery devices used before HFNC therapy (Table 1).

HFNC - high flow nasal cannula; MV - mechanical ventilation; ICU - intensive care unit.

**Table 1:** Clinical data of patients admitted to intensive care units who received high-flow nasal cannula oxygen therapy

		Total (n = 444)	Intubated (n = 261)	Nonintubated (n = 183)	p value
Age (years)		61 (52 - 75)	59 (49 - 73)	65 (54 - 77)	0.001
Male sex		292 (65.7)	176 (67.4)	116 (63.3)	0.38
SAPS 3		43 (37 - 50)	44 (37 - 52)	42 (36 - 47)	< 0.001
Comorbidities					
	Cardiovascular	89 (20)	55 (21)	34 (18.5)	0.51
	Respiratory	24 (5.4)	16 (6.1)	8 (4.3)	0.41
	Neurological	7 (1.5)	5 (1.9)	2 (1)	0.49
	Gastroenterological	9 (2)	6 (2.2)	3 (1.6)	0.62
	Renal/metabolic	87 (19.5)	56 (21.4)	31 (16.6)	0.23
	Neoplasm	29 (6.5)	21 (8)	8 (4.3)	0.12
	Immunological	7 (1.5)	5 (1.9)	2 (1)	0.49
Charlson		3 (2 - 4)	3 (1 - 4)	3 (2 - 4)	0.45
Device before HFNC					
	Nasal catheter	74 (16.6)	40 (15.3)	34 (18.5)	0.36
	Face tent/macronebulizer	3 (0.6)	2 (0.7)	1 (0.5)	0.78
	Reservoir mask	357 (80.4)	215 (82.3)	142 (77.5)	0.21
	Venturi mask	5 (1.1)	1 (0.3)	4 (2.1)	0.07
Awake prone positioning		251 (56.5)	145 (55.5)	106 (57.9)	0.62
Noninvasive ventilation		288 (64.8)	181 (69.3)	107 (58.4)	0.01

SAPS 3 - Simplified Acute Physiology Score 3; HFNC - high-flow nasal cannula. P value: comparison between intubated and nonintubated patients. The results are presented as the medians (interquartile ranges) or n (%).

During follow-up, the intubated patients exhibited longer ICU (p < 0.001) and hospital (p < 0.001) stays and greater ICU and hospital mortality (p < 0.001) (Table 2). All deaths occurred after intubation. Among the patients who required invasive ventilatory support, 245 (94.5%) were intubated within 7 days, and 184 (71.0%) were intubated within 48 hours after HFNC therapy started (Supplementary Material - Figure 1S). The duration of MV was 10 days (5 - 21) (Supplementary Material - Table 1S).

**Table 2:** Follow-up data of patients admitted to intensive care units who received high-flow nasal cannula oxygen therapy

		Total (n = 444)	Intubated (n = 261)	Nonintubated (n = 183)	p value
ICU length of stay (days)		11 (7 - 21)	18 (11-28)	7 (5 - 10)	< 0.001
Hospital length of stay (days)		19 (12 - 31)	25 (15 - 40)	14 (10 - 21)	< 0.001
Hospital death		123 (27.7)	123 (47.1)	0 (0)	< 0.001
Cause of death					
	Refractory hypoxemia	60 (48.7)	60 (48.7)	0 (0)	-
	Refractory shock	38 (30.8)	38 (30.8)	0 (0)	-
	Multiple organ dysfunction	21 (17)	21 (17)	0 (0)	-
	Other	4 (3.2)	4 (3.2)	0 (0)	-

ICU - intensive care unit; p value: comparison between intubated and nonintubated patients. The results are presented as the medians (interquartile ranges) or n (%).

### Respiratory parameters

By comparing basal vital signs, SpO_2_, parameters adjusted during HFNC therapy, and the ROX index between intubated and nonintubated patients at different durations of HFNC therapy, we observed that the respiratory rate was similar between groups from baseline to the 6th hour but was higher in the first 12 hours (24 [21 - 27] *versus* 22 [19 - 25]; p = 0.002) and 24 hours (25 [22 - 27] *versus* 22 [19 - 24]; p < 0.001) after HFNC therapy started. Intubated patients also had significantly higher flow and FiO_2_ and a lower ROX index at all time points, while the SpO_2_ was lower after the 6th hour (Supplementary Material - Table 2S).

### Accuracy of the different parameters

**Primary outcome:** an analysis of the AUROCs revealed a greater capacity to discriminate between successful and failed HFNC therapy within 7 days for ROX-24 h (AUROC 0.80; 95%CI 0.76 - 0.84), followed by SpO_2_/FiO_2_ at 24 hours (AUROC 0.76; 95%CI 0.71 - 0.80). The median interval between HFNC therapy initiation and intubation was 24 hours (24 - 72), and the most accurate predictor of intubation measured before 24 hours was ROX-12 h (AUROC 0.75; 95%CI 0.70 - 0.79), followed by ROX-6 h (AUROC 0.71; 95%CI 0.67 - 0.76). Using the best cutoff value of each of these parameters, ROX-24 h ≤ 5.96 showed 80.6% sensitivity and 68.9% specificity for predicting the need for intubation within 7 days compared to SpO_2_/FiO_2_ ≤ 129 at 24 hours (sensitivity: 71.0%, specificity: 70.2%), ROX-12 h ≤ 5.54 (sensitivity: 67.3%, specificity: 72.8%), and ROX-6 h ≤ 6.08 (sensitivity: 69.4%, specificity: 65.9). When ROX-12 h ≤ 4.88 (reported as the cutoff value of the ROX index in non-COVID-19 patients)^(19,20)^ was used arbitrarily, the sensitivity was 57% (95%CI 47 - 72), and the specificity was 78% (95%CI 72 - 84; Youden index J = 0.40; Z statistic = 10.1). The sensitivity, specificity and positive and negative predictive values for the different cutoffs of each parameter are shown in table 3 and table 3S (Supplementary Material).

**Table 3:** Analysis of the area under the receiver operating characteristic curve and cutoff values of the respiratory oxygenation index for identifying the success or failure of high-flow nasal cannula therapy and the need for orotracheal intubation within 7 days and 48 hours

Orotracheal intubation within 7 days								
	AUROC	95%CI	p value	Cutoff	Sensitivity	Specificity	Youden index J	Z statistic
ROX-baseline	0.69	0.65 - 0.74	< 0.001	≤ 5.35	68.1	64.8	0.32	7.8
ROX-2 h	0.69	0.64 - 0.73	< 0.001	≤ 5.57	67	67.1	0.34	7.5
ROX-4 h	0.68	0.64 - 0.73	< 0.001	≤ 5.82	67.8	66.6	0.34	7.2
ROX-6 h	0.71	0.67 - 0.76	< 0.001	≤ 6.08	69.4	65.9	0.35	8.5
ROX-12 h	0.75	0.70 - 0.79	< 0.001	≤ 5.54	67.3	72.8	0.4	10.1
ROX-24 h	0.8	0.76 - 0.84	< 0.001	≤ 5.96	80.6	68.9	0.49	13.3
**Orotracheal intubation within 48 hours**								
	**AUROC**	**95%CI**	**p value**	**Cutoff**	**Sensitivity**	**Specificity**	**Youden index J**	**Z statistic**
ROX-baseline	0.65	0.61 - 0.7	< 0.001	≤ 5.16	66.1	59.3	0.25	5.9
ROX-2 h	0.67	0.62 - 0.71	< 0.001	≤ 5.57	67.9	59.6	0.27	6.5
ROX-4 h	0.65	0.60 -0.69	< 0.001	≤ 5.82	68.4	59.1	0.27	5.7
ROX-6 h	0.69	0.64 - 0.73	< 0.001	≤ 5.17	60.9	71.2	0.32	7.3
ROX-12 h	0.73	0.68 - 0.77	< 0.001	≤ 5.54	71.5	65.4	0.37	8.9
ROX-24 h	0.78	0.73 - 0.82	< 0.001	≤ 4.84	72.5	76.5	0.49	10.2

AUROC - area under the receiver operating characteristic curve; 95%CI - 95% confidence interval; ROX - respiratory oxygenation index.

**Secondary outcome:** in general, an analysis of intubation within 48 hours revealed slightly lower AUROCs than those observed for the 7-day analysis (Table 3 and Table 3S - Supplementary Material).

### Sensitivity analysis

For patients who were intubated within 24 hours, the ROX-12 h had an AUROC of 0.74 (95%CI 0.69 - 0.78). For a ROX-12 h ≤ 5.26, the sensitivity was 73.1%, and the specificity was 66.7%. An important reduction in the accuracy of the variables analyzed was observed in the population intubated after 24 hours of HFNC oxygen therapy (Table 4S - Supplementary Material).

### Comparison of ROC curves

**Figure 2:**
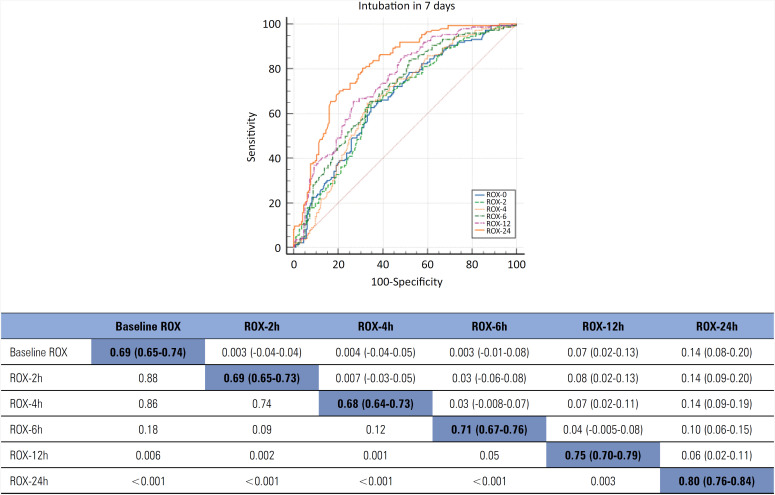
Area under the receiver operating characteristic curve and 95% confidence intervals for respiratory oxygenation index measurements at different time intervals from the start of high-flow nasal cannula therapy (baseline, 2, 4, 6, 12 and 24 hours) and its association with orotracheal intubation within 7 days.

A comparison between the AUROCs of the ROX indices obtained at the different time points (baseline and 2, 4, 6, 12 and 24 hours) revealed significant differences in favor of measurements performed 12 and 24 hours after the initiation of HFNC therapy compared to measurements collected for up to 6 hours for both the primary and secondary outcomes (Figure 2 and Figure 2S and Table 5S in the Supplementary Material). For the subgroup of patients included in the sensitivity analysis, the ROX-12 h measurements differed from those performed up to 6 hours after HFNC therapy started (Figure 2S - Supplementary Material).

The data for the dark blue cells on the central diagonal refer to the areas under the receiver operating characteristic curves of the respiratory oxygenation index measured at different times after the installation of the high-flow nasal cannula. The p values for comparisons between respiratory oxygenation index measurement time points are shown below the area under the receiver operating characteristic curve. Differences between the areas under the curves at different time points are shown above the area under the receiver operating characteristic curve.

ROX - respiratory oxygenation index.

### Kaplan-Meier curves

**Figure 3:**
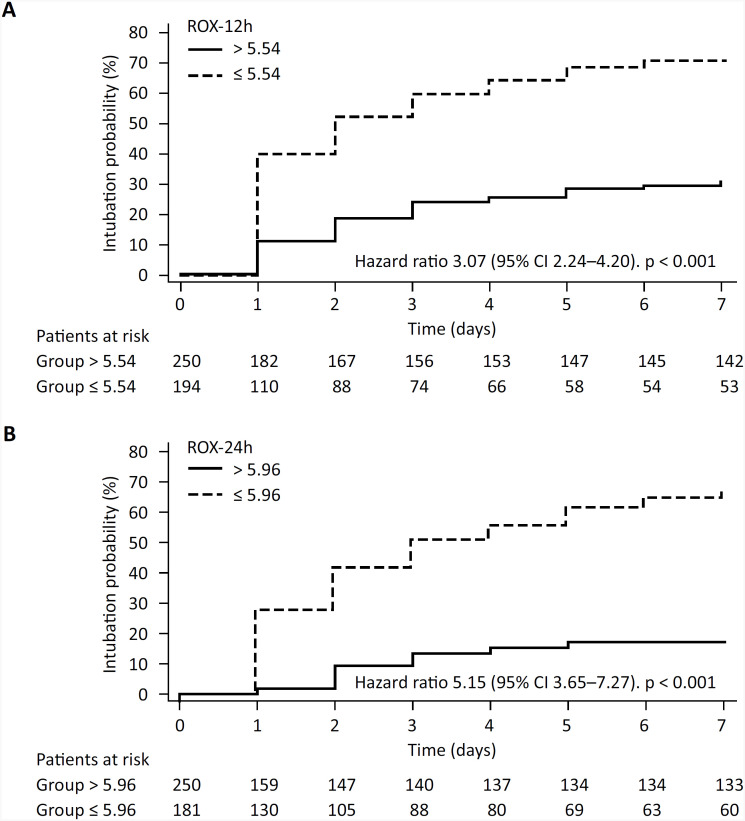
Kaplan-Meier curves.

The Kaplan-Meier curves showed a greater probability of orotracheal intubation within 7 days among patients with ROX-12 h ≤ 5.54 (hazard ratio 3.07; 95%CI 2.24 - 4.20) and ROX-24 h ≤ 5.96 (hazard ratio 5.15; 95%CI 3.65 - 7.27) (Figure 3).

(A) Probability of orotracheal intubation within 7 days in patients with an respiratory oxygenation index 12 h > 5.54 *versus* patients with an respiratory oxygenation index 12 h ≤ 5.54 and (B) probability of orotracheal intubation within 7 days in patients with an respiratory oxygenation index 24 h > 5.96 *versus* patients with an respiratory oxygenation index 24 h x ≤ 5.96.

ROX - respiratory oxygenation index; 95%CI - 95% confidence interval.

## DISCUSSION

In this retrospective cohort study, we observed that the ROX index was a good predictor for identifying the need for intubation in COVID-19 patients in the ICU who received HFNC therapy. More precisely, a ROX index ≤ 5.96 after 24 hours of HFNC supportive therapy was more accurate for identifying the risk of intubation within 7 days. Although ROX-12 h ≤ 5.54 did not indicate the need for intubation with the same accuracy, its discriminatory capacity was moderate, similar to the findings of other authors.^(20-23)^

During the COVID-19 pandemic, HFNC therapy was widely used after doubts and fears regarding the safety of the health team had been overcome,^(28,29)^ and HFNC therapy was found to be a very interesting alternative to noninvasive ventilatory support in hypoxemic patients with severe acute respiratory syndrome due to COVID-19.^(30-37)^ Oxygen therapy with a HFNC significantly reduced the need for MV and the time of clinical recovery compared to conventional oxygen therapy, with no impact on mortality or the length of ICU stay.^(7-9,38)^

Early identification of the need for intubation in patients receiving noninvasive ventilatory support is associated with the patient prognosis.^(12)^ However, this identification is challenging because of the inaccuracy of the individual use of common clinical parameters, such as the respiratory rate, oxygen concentration, and SpO_2_.^(18,19)^ Although evidence indicates that a HFNC can reduce the inspiratory effort of patients with acute respiratory failure due to COVID-19,^(39)^ uncertainties persist regarding the ideal time when invasive MV should be started, as well as regarding the relative risks of lung injury self-inflicted by the patient *versus* ventilator-induced lung injury.^(40-44)^ Knowing the predictive value of the ROX index in the prepandemic scenario, some authors have evaluated the potential of this parameter in monitoring COVID-19 patients to identify the failure of HFNC therapy or noninvasive mask ventilation and have found good accuracy of this index in predicting the need for intubation.^(18-22)^ Because HFNC therapy has been widely used during the COVID-19 pandemic in the ICU and outside the ICU, the ROX index has become an important tool for identifying patients who may deteriorate and need ICU admission and MV.^(15)^

With the aim of evaluating the predictive performance of the ROX index for successful weaning from HFNC therapy in pneumonia patients with acute hypoxemic respiratory failure, a systematic review and meta-analysis of thirteen observational studies involving 1,751 patients revealed that the ROX index, measured within 12 hours after HFNC therapy initiation, exhibited good performance at predicting successful weaning from HFNC therapy, with mean and median cutoff values of the ROX index of 4.8 (95%CI 4.2 - 5.4) and 5.3 (95%CI 4.2 - 5.5), respectively.^(16)^ Similar results were reported by other authors.^(17)^ When analyzing the accuracy of the ROX index in patients with acute respiratory failure due to COVID-19, another systematic review and meta-analysis of eight studies involving 1,301 patients indicated good discriminatory power of the ROX index in identifying the failure of HFNC therapy (summary AUROC 0.81; 95%CI 0.77 - 0.84).^(23)^

We observed acceptable accuracy of the ROX-12 h index in discriminating patients with respiratory failure due to COVID-19 who may progress to HFNC therapy failure (AUROC = 0.75), which was supported by the sensitivity analysis. The median interval between HFNC therapy initiation and intubation was 24 hours, indicating that half of the patients underwent orotracheal intubation within 24 hours. Therefore, the ROX-24 h index was obtained just before intubation or after intubation in half of the patients. We must emphasize that the ROX index was determined during HFNC therapy, and patients who were intubated after 12 hours of HFNC therapy were not considered in the analysis of the ROX-24 h index. Roca et al.^(18,19)^ reported very similar results regarding the predictive capacity of the ROX index measured 12 hours after the start of HFNC therapy in patients with hypoxemic respiratory failure due to community pneumonia. Furthermore, these authors found a greater accuracy of the ROX index in predicting intubation due to failure of HFNC therapy compared to other commonly used variables, such as the respiratory rate, oxygen flow and SpO_2_, as also observed in the present study.

Within this context, although the ROX-24 h index is more accurate (AUROC = 0.80) than the commonly used parameters, identifying the need for intubation only 24 hours after starting HFNC therapy seems to be late and may negatively affect patient prognosis.^(12)^ We found that the rate of failure and need for intubation were still significant within 7 days after the start of HFNC therapy, with 75 (28.7%) of the patients intubated after 48 hours and up to 7 days (Figure 1S - Supplementary Material). These findings suggest that the ROX index might be used to identify patients who have a more severe respiratory disease, who may have an unfavorable outcome and who deserve greater surveillance and monitoring in an intensive care unit. However, the ROX index can reflect a specific moment in time instead of the clinical evolution of the patient, and these parameters can easily vary throughout the day or in different clinical situations (fever, mobilization, fatigue, pain, acidosis, and hypotension). This result suggests that other parameters, such as neurological deterioration, work of breathing, mental status alterations, agitation, drowsiness, and stupor, should not be ignored. Regarding the external validity of our findings, the database of this multicenter study included critically ill adult patients in many Brazilian states who were managed in different settings. Additionally, all patients had a confirmed diagnosis of COVID-19 and were subjected to HFNC oxygen therapy with similar criteria for acute respiratory failure.

Our study has several limitations. Although it is a multicenter study involving institutions from different places and with different characteristics, as well as with a history of participation in multicenter studies, it has all the limitations inherent to its retrospective design. The selection of the time with the best AUROC and of the cutoff value of the ROX index with the best sensitivity were post hoc procedures and may reflect random errors; these procedures have not been validated in independent samples. The participating centers did not use a single protocol with preestablished criteria for the use of HFNC therapy; hence, we may have inadvertently excluded patients who met the criteria for the use of this therapy or even included others who did not meet the criteria. Likewise, the criteria for defining therapeutic failure and the indication for intubation have not been standardized previously; however, the participating centers did not lack resources that could delay intubation. Different brands of HFNC devices with different characteristics were used for oxygen therapy. In some of the participating centers, HFNC therapy started to be used during the pandemic, which indicates a short period of familiarization with the method. Some data were not recorded, including the interval between the diagnosis of respiratory failure and the start of HFNC therapy; in addition, the duration of noninvasive ventilation, which was used before and during HFNC therapy, was not recorded or standardized.

## CONCLUSION

Our results suggest that the respiratory oxygenation index can help identify patients who will progress to failure of high-flow nasal cannula supportive therapy. This index is more accurate than commonly used parameters, such as the respiratory rate or peripheral oxygen saturation. These findings are important for assisting intensive care and emergency care professionals in the early identification of these patients and avoiding delays in intubation.
