# Supplementary material for: The respiratory oxygenation index for identifying the risk of orotracheal intubation in COVID-19 patients receiving high-flow nasal cannula oxygen
Source: Crit Care Sci. 2024 Jun 14;36:e20240203en. doi: 10.62675/2965-2774.20240203-en (PMC11208043; doi:10.62675/2965-2774.20240203-en)
Supplement: Supplementary file 1 [file 2965-2774-ccsci-36-e20240203en-Suppl01.pdf]

Critical Care Science
https://doi.org/10.62675/2965-2774.20240203-en

SUPPLEMENTARY MATERIAL

The respiratory oxygenation index for identifying the risk of orotracheal intubation in COVID-19 patients receiving high-flow nasal cannula oxygen

Aline Braz Pereira[1], Felipe Dal Pizzol[2], Viviane Cordeiro Veiga[3], Leandro Utino Taniguchi[4], Aline Finoti Misquita[3], Gustavo Augusto Couto Carvalho[3], Ligia Maria Coscrato Junqueira Silva[3], Michelli Marcela Dadam[5], Ruthy Perotto Fernandes[1], Israel Silva Maia[6], Cassio Luis Zandonai[6], Alexandre Biasi Cavalcanti[7], Marcelo Luz Pereira Romano[7], Glauco Adrieno Westphal[1] on behalf of the Brazilian Research in Intensive Care Network (BRICNet)

Table 1S - Data of patients using a high-flow nasal cannula who required orotracheal intubation and mechanical ventilation

Orotracheal intubation	259 (58.5)
Orotracheal intubation within 48 hours	184 (71)
Orotracheal intubation within 7 days	245 (94.5)
HFNC data at the time of intubation	
RR (bpm)	28 (24 - 32)
SpO_2 (%)	88 (86 - 91)
Flow (L/minute)	60 (60 - 60)
FiO_2 (%)	100 (90 - 100)
ROX index	3.2 (2.8 - 4)
Extubation	131 (50.5)
Tracheostomy	54 (20.8)
Interval between HFNC start and OTI (hours)	24 (24 - 72)
Duration of MV (days)	10 (5 - 21)

HFNC - high-flow nasal cannula; RR - respiratory rate (breaths per minute); SpO_2 - peripheral oxygen saturation; FiO_2 - inspired fraction of oxygen; ROX - respiratory oxygenation index; OTI - orotracheal intubation; MV - mechanical ventilation. Results are expressed in n (%) or median (interquartile range).

This is an open access article under the CC BY license (https://creativecommons.org/licenses/by/4.0/).

Table 2S - Clinical data, high-flow nasal cannula parameters and respiratory oxygenation index of patients admitted to intensive care units

	Total (n = 442)	Intubated (n = 259)	Non-intubated (n = 183)	p value
**Baseline**				
MAP (mmHg)	93 (83 - 103)	93 (83 - 102)	93 (84 - 103)	0.11
HR (bpm)	85 (75 - 90)	85 (76 - 91)	83 (74 - 90)	0.20
RR (bpm)	24 (21 - 28)	25 (21 - 28)	24 (21 - 28)	0.27
SpO_2 (%)	93 (90 - 95)	92 (90 - 95)	94 (91 - 95)	0.16
Flow (L/minute)	60 (40 - 60)	60 (40 - 60)	55 (40 - 60)	0.02
FiO_2 (%)	70 (60 - 100)	80 (63 - 100)	60 (50 - 80)	< 0.001
ROX index	5.0 (3.8 - 7.1)	4.5 (3.6 - 6.2)	6.3 (4.4 - 8.8)	< 0.001
**2nd hour**				
RR (bpm)	23 (20 - 26)	23 (20 - 27)	22 (20 - 25)	0.21
SpO_2 (%)	93 (91 - 95)	92 (90 - 95)	94 (92 - 96)	0.02
Flow (L/minute)	60 (40 - 60)	60 (40 - 60)	60 (40 - 60)	0.03
FiO_2 (%)	70 (57 - 100)	80 (60 - 100)	60 (50 - 80)	< 0.001
ROX index	5.5 (4.2 - 7.6)	4.8 (4 - 6.5)	6.6 (4.7 - 9.3)	< 0.001
**4th hour**				
RR (bpm)	23 (20 - 26)	23 (20 - 26)	22 (20 - 25)	0.12
SpO_2 (%)	93 (91 - 95)	93 (91 - 95)	94 (92 - 96)	0.29
Flow (L/minute)	60 (40 - 60)	60 (40 - 60)	60 (40 - 60)	0.02
FiO_2 (%)	70 (55 - 94)	80 (60 - 100)	60 (50 - 80)	< 0.001
ROX index	5.7 (4.4 - 7.8)	5.0 (4 - 6.5)	7.0 (5.3 - 9.5)	< 0.001
**6th hour**				
RR (bpm)	23 (20 - 26)	24 (20 - 27)	22 (20 - 25)	0.11
SpO_2 (%)	93 (91 - 95)	92 (90 - 94)	94 (92 - 95)	0.02
Flow (L/minute)	60 (40 - 60)	60 (45 - 60)	50 (40 - 60)	0.008
FiO_2 (%)	70 (52 - 90)	80 (60 - 100)	60 (45 - 75)	< 0.001
ROX index	5.8 (4.4 - 8.1)	4.8 (4 - 6.5)	7.5 (5.4 - 9.6)	< 0.001
**12th hour**				
RR (bpm)	23 (20 - 26)	24 (21 - 27)	22 (19 - 25)	0.002
SpO_2 (%)	93 (90 - 95)	92 (90 - 94)	93 (92 - 96)	0.02
Flow (L/minute)	60 (44 - 60)	60 (50 - 60)	50 (40 - 60)	0.004
FiO_2 (%)	70 (50 - 90)	80 (64 - 100)	60 (45 - 70)	< 0.001
ROX index	5.7 (4.3 - 8.1)	4.7 (3.8 - 6.3)	7.3 (5.5 - 10)	< 0.001
**24th hour**				
RR (bpm)	23 (20 - 26)	25 (22 - 27)	22 (19 - 24)	< 0.001
SpO_2 (%)	93 (91 - 95)	92 (90 - 94)	94 (92 - 95)	< 0.001
Flow (L/minute)	60 (40 - 60)	60 (50 - 60)	55 (40 - 60)	< 0.001
FiO_2 (%)	70 (50 - 90)	90 (70 - 100)	60 (40 - 71)	< 0.001
ROX index	5.7 (4.1 - 8.1)	4.5 (3.6 - 5.7)	7.6 (5.6 - 10)	< 0.001

MAP - mean arterial pressure; HR - heart hate (beats per minute); RR - respiratory rate (breaths per minute); SpO_2 - peripheral oxygen saturation; FiO_2 - inspired fraction of oxygen; ROX - respiratory oxygenation index; p value: comparison between intubated and non-intubated patients. Results are expressed in median (interquartile range).

Table 3S - Analysis of area under the receiver operating characteristics and cut-off values of respiratory rate, SpO_2, flow and SpO_2/FiO_2 for identifying success or failure of high-flow nasal cannula therapy and need for orotracheal intubation within 7 days and 48 hours

Orotracheal intubation within 7 days					
	RR	SpO_2	Flow	FiO_2	SpO_2/FiO_2
**Start**					
AUROC	0.56	0.57	0.54	0.71	0.69
95%CI	0.51 - 0.6	0.52 - 0.61	0.49 - 0.59	0.67 - 0.75	0.65 - 0.73
p value	0.02	0.008	0.08	< 0.001	< 0.001
Cut-off	> 24	≤ 93	> 40	> 71	≤ 132
Sensitivity	51.8	64.9	72.6	62.8	66.5
Specificity	60.8	50.2	35.1	71.3	62.8
Youden index J	0.12	0.15	0.07	0.34	0.29
Z statistic	2.2	2.6	1.7	8.8	7.8
**2 hours**					
AUROC	0.55	0.61	0.54	0.7	0.68
95%CI	0.51 - 0.6	0.56 - 0.65	0.49 - 0.59	0.65 - 0.74	0.64 - 0.72
p value	0.03	< 0.001	0.08	< 0.001	< 0.001
Cut-off	> 24	≤ 92	> 55	> 74	≤ 125
Sensitivity	37.8	51	59.2	62.1	61.7
Specificity	71.7	70.2	48.9	69.7	66.6
Youden index J	0.09	0.2	0.08	0.3	0.28
Z statistic	2.1	4.2	1.7	8.1	7.3
**4 hours**					
AUROC	0.54	0.57	0.53	0.7	0.68
95%CI	0.49 - 0.59	0.53 - 0.62	0.48 - 0.58	0.66 - 0.75	0.64 - 0.73
p value	0.09	0.004	0.17	< 0.001	< 0.001
Cut-off	> 21	≤ 93	> 44	> 66	≤ 134
Sensitivity	67.8	60	73.9	70.4	68.1
Specificity	41.9	53	33.3	61.6	62.1
Youden index J	0.09	0.13	0.07	0.32	0.3
Z statistic	1.6	2.8	1.3	8.3	7.1
**6 hours**					
AUROC	0.56	0.59	0.54	0.72	0.69
95%CI	0.51 - 0.6	0.54 - 0.64	0.5 - 0.59	0.67 - 0.76	0.65 - 0.74
p value	0.03	< 0.001	0.05	< 0.001	< 0.001
Cut-off	> 24	≤ 91	> 55	> 65	≤151
Sensitivity	41.4	40.2	60.1	72.3	77.1
Specificity	70.7	77.1	48.9	63.6	56.8
Youden index J	0.12	0.17	0.09	0.37	0.34
Z statistic	2.1	3.5	1.9	9	7.6
**12 hours**					
AUROC	0.61	0.61	0.56	0.73	0.73
95%CI	0.56 - 0.66	0.56 - 0.66	0.51 - 0.61	0.68 - 0.77	0.68 - 0.77
p value	< 0.001	<0.001	0.01	< 0.001	< 0.001
Cut-off	> 20	≤ 90	> 50	> 65	≤ 146

Continue...

...continuation

Orotracheal intubation within 7 days					
	RR	SpO_2	Flow	FiO_2	SpO_2/FiO_2
Sensitivity	78.4	37.8	63.1	75.2	77.7
Specificity	40.5	85.1	50.7	67.6	62.3
Youden index J	0.18	0.23	0.13	0.42	0.4
Z statistic	3.8	4.1	2.4	9.4	9.1
**24 hours**					
AUROC	0.69	0.65	0.59	0.76	0.76
95%CI	0.64 - 0.74	0.6 - 0.7	0.54 - 0.64	0.71 - 0.8	0.71 - 0.8
p value	< 0.001	< 0.001	< 0.001	< 0.001	< 0.001
Cut-off	> 23	≤ 91	> 45	> 75	≤ 129
Sensitivity	67.5	43.3	80.7	67.5	71
Specificity	68.3	82.3	35.7	74.6	70.2
Youden index J	0.35	0.25	0.16	0.42	0.41
Z statistic	6.6	5.1	3.4	10.5	10.4
**Orotracheal intubation within 48 hours**					
	RR	SpO_2	Flow	FiO_2	SpO_2/FiO_2
**Start**					
AUROC	0.55	0.59	0.54	0.65	0.64
95%CI	0.5 - 0.6	0.55 - 0.64	0.5 - 0.59	0.61 - 0.7	0.6 - 0.69
p value	0.04	< 0.001	0.05	< 0.001	< 0.001
Cut-off	> 25	≤ 93	> 55	> 71	≤ 131
Sensitivity	44.2	68.8	59.5	62.3	67.2
Specificity	67.0	49.4	49	62.8	56.7
Youden index J	0.11	0.18	0.08	0.25	0.23
Z statistic	2	3.6	1.9	6.1	5.6
**2 hours**					
AUROC	0.57	0.65	0.55	0.65	0.63
95% CI	0.52 - 0.62	0.6 - 0.69	0.5 - 0.6	0.6 - 0.69	0.58 - 0.67
p value	0.006	< 0.001	0.03	< 0.001	< 0.001
Cut-off	> 24	≤ 92	> 40	> 71	≤ 125
Sensitivity	40.3	56.3	75.1	61.8	60.9
Specificity	71.1	68.8	33.8	61.1	59.4
Youden index J	0.11	0.25	0.08	0.23	0.2
Z statistic	2.7	5.7	2.1	5.8	5
**4 hours**					
AUROC	0.54	0.6	0.55	0.66	0.64
95%CI	0.49 - 0.59	0.55 - 0.64	0.51 - 0.6	0.61 - 0.71	0.6 - 0.69
p value	0.13	< 0.001	0.02	< 0.001	< 0.001
Cut-off	> 21	≤ 93	> 40	> 70	≤ 143
Sensitivity	69	64.3	77.1	61.9	72.9
Specificity	40.4	52.9	33	63.4	51.9
Youden index J	0.09	0.17	0.1	0.25	0.24
Z statistic	1.5	3.6	2.3	6.3	5.6

Continue...

...continuation

Orotracheal intubation within 48 hours					
	RR	SpO_2	Flow	FiO_2	SpO_2/FiO_2
**6 hours**					
AUROC	0.57	0.63	0.58	0.68	0.68
95%CI	0.52 - 0.61	0.58 - 0.68	0.53 - 0.62	0.64 - 0.73	0.64 - 0.73
p value	0.01	< 0.001	0.001	< 0.001	< 0.001
Cut-off	> 22	≤ 91	> 55	> 60	≤ 151
Sensitivity	62.4	47.4	64.3	75.1	80.1
Specificity	50	77.4	49.4	52.7	51.6
Youden index J	0.12	0.24	0.13	0.27	0.31
Z statistic	2.4	4.6	3.1	7.3	7.1
**12 hours**					
AUROC	0.6	0.65	0.59	0.7	0.69
95%CI	0.55 - 0.65	0.6 - 0.7	0.54 - 0.64	0.65 - 0.75	0.65 - 0.74
p value	< 0.001	< 0.001	< 0.001	< 0.001	< 0.001
Cut-off	> 21	≤ 92	> 40	> 70	≤ 150
Sensitivity	79.2	63.8	86.1	65.3	84.2
Specificity	41.1	62.3	30.5	67	53.9
Youden index J	0.2	0.26	0.16	0.32	0.38
Z statistic	3.7	5.3	3.8	7.7	7.5
**24 hours**					
AUROC	0.66	0.67	0.64	0.73	0.74
95%CI	0.6 - 0.71	0.62 - 0.72	0.58 - 0.69	0.68 - 0.77	0.69 - 0.78
p value	< 0.001	< 0.001	< 0.001	< 0.001	< 0.001
Cut-off	> 23	≤ 91	> 55	> 70	≤ 131
Sensitivity	69.5	49.4	75	72.8	76.1
Specificity	60.7	78.5	49.6	64.6	60.6
Youden index J	0.3	0.28	0.24	0.37	0.3
Z statistic	5	5	5.2	8.1	8.7

AUROC - area under the receiver operating characteristic; 95%CI - 95% confidence interval; RR - respiratory rate; SpO_2 - peripheral oxygen saturation; FiO_2 - inspired fraction of oxygen.

Table 4S - Analysis of area under the receiver operating characteristics and cut-off values of respiratory rate, SpO_2, flow, SpO_2/FiO_2 and respiratory oxygenation index for identifying success or failure of high-flow nasal cannula therapy and need for orotracheal intubation within 24 hours and within > 24 hours to 7 days

Orotracheal intubation within 24 hours						
	RR	SpO_2	Flow	FiO_2	SpO_2/FiO_2	ROX index
**Start**						
AUROC	0.54	0.59	0.54	0.66	0.63	0.65
95%CI	0.49 - 0.59	0.55 - 0.64	0.5 - 0.59	0.62 - 0.71	0.58 - 0.67	0.58 - 0.69
p value	0.13	< 0.001	0.05	< 0.001	< 0.001	< 0.001
Cut-off	> 19	≤ 93	> 55	> 60	≤ 169	≤ 5.16
Sensitivity	93.2	70.4	60.4	79.1	91.2	69.1
Specificity	15.2	48.1	48.4	47.1	29.1	57.9
Youden index J	0.08	0.18	0.08	0.26	0.2	0.27
Z statistic	1.5	3.54	1.9	6.59	4.87	6.04
**2 hours**						
AUROC	0.55	0.69	0.56	0.67	0.61	0.68
95%CI	0.51 - 0.6	0.64 - 0.73	0.51 - 0.6	0.62 - 0.71	0.56 - 0.66	0.64 - 0.73
p value	0.04	< 0.001	0.01	< 0.001	< 0.001	< 0.001
Cut-off	> 29	≤ 93	> 55	> 75	≤ 180	≤ 5.88
Sensitivity	20.4	75.5	62.5	64.6	92.5	76.1
Specificity	89.4	55.4	47.9	62.2	26.2	55.4
Youden index J	0.09	0.3	0.1	0.26	0.18	0.31
Z statistic	1.98	7.14	2.38	6.75	4.16	7.14
**4 hours**						
AUROC	0.56	0.61	0.55	0.69	0.63	0.69
95%CI	0.51 - 0.61	0.56 - 0.66	0.51 - 0.6	0.65 - 0.73	0.58 - 0.67	0.64 - 0.73
p value	0.02	< 0.001	0.02	< 0.001	< 0.001	< 0.001
Cut-off	> 20	≤ 91	> 55	> 75	≤ 172	≤ 5.81
Sensitivity	78.6	41.1	61.7	64.7	92.1	74.2
Specificity	34.2	79.1	47.6	65.4	30.6	58.9
Youden index J	0.12	0.2	0.09	0.3	0.22	0.33
Z statistic	2.19	3.8	2.2	7.6	4.84	7.25
**6 hours**						
AUROC	0.55	0.64	0.57	0.71	0.66	0.7
95%CI	0.5 - 0.6	0.59 - 0.69	0.53 - 0.62	0.67 - 0.75	0.61 - 0.71	0.65 - 0.74
p value	0.05	< 0.001	0.002	< 0.001	< 0.001	< 0.001
Cut-off	> 22	≤ 92	> 55	> 60	≤ 151	≤ 5.17
Sensitivity	63.1	60.8	65.5	81.1	79.7	66.6
Specificity	48.8	64.8	48.2	51.8	47.7	69.6
Youden index J	0.11	0.25	0.13	0.33	0.27	0.36
Z statistic	1.89	4.62	2.99	8.37	5.96	7.39
**12 hours**						
AUROC	0.62	0.67	0.57	0.71	0.65	0.74
95%CI	0.57 - 0.67	0.62 - 0.71	0.52 - 0.62	0.66 - 0.75	0.6 - 0.7	0.69 - 0.78
p value	< 0.001	< 0.001	0.005	< 0.001	< 0.001	< 0.001

Continue...

...continuation

Orotracheal intubation within 24 hours						
	**RR**	**SpO_2**	**Flow**	**FiO_2**	**SpO_2/FiO_2**	**ROX index**
Cut-off	> 21	≤ 91	> 40	> 67	≤ 150	≤ 5.26
Sensitivity	82.8	56.9	87.2	80.6	81.3	73.1
Specificity	39.7	74.6	28.7	55.4	48.7	66.7
Youden index J	0.22	0.31	0.15	0.36	0.3	0.39
Z statistic	4.03	5.24	2.77	7.71	5.51	9.02
**Orotracheal intubation within > 24 hours to 7 days**						
	**RR**	**SpO_2**	**Flow**	**FiO_2**	**SpO_2/FiO_2**	**ROX index**
**Start**						
AUROC	0.52	0.52	0.5	0.58	0.6	0.57
95%CI	0.48 - 0.57	0.47 - 0.57	0.45 - 0.55	0.53 - 0.63	0.56 - 0.65	0.52 - 0.61
p value	0.41	0.45	0.87	0.006	< 0.001	0.02
Cut-off	> 24	> 94	> 48	> 71	≤ 118	≤ 5.53
Sensitivity	53	36.7	70.4	60.2	60.2	66.3
Specificity	55.7	74.8	34.6	56	59.5	46.5
Youden index J	0.08	0.11	0.05	0.16	0.19	0.12
Z statistic	0.81	0.74	0.15	2.7	3.44	2.24
**2 hours**						
AUROC	0.51	0.58	0.5	0.56	0.61	0.53
95%CI	0.46 - 0.55	0.53 - 0.62	0.46 - 0.55	0.51 - 0.61	0.57 - 0.66	0.48 - 0.58
p value	0.76	0.01	0.77	0.03	< 0.001	0.27
Cut-off	> 23	> 94	≤ 38	> 74	≤ 132	≤ 8.34
Sensitivity	48.9	44.9	15.3	58.1	72.1	90.8
Specificity	56.8	70.2	88.9	55.1	50.5	23
Youden index J	0.05	0.15	0.04	0.13	0.22	0.13
Z statistic	0.29	2.41	0.28	2.06	3.74	1.08
**4 hours**						
AUROC	0.51	0.51	0.51	0.55	0.59	0.53
95%CI	0.46 - 0.56	0.47 - 0.57	0.46 - 0.56	0.5 - 0.6	0.55 - 0.64	0.48 - 0.58
p value	0.72	0.5	0.59	0.08	0.001	0.25
Cut-off	≤ 24	> 91	≤ 35	> 66	≤ 134	≤ 9.29
Sensitivity	72.9	78.1	15.6	64.5	70.2	92.7
Specificity	33.7	28.9	89.4	46.9	50.3	18.3
Youden index J	0.06	0.07	0.05	0.11	0.2	0.11
Z statistic	0.35	0.67	0.53	1.72	3.14	1.13
**6 hours**						
AUROC	0.52	0.52	0.51	0.56	0.58	0.56
95%CI	0.47 - 0.57	0.47 - 0.57	0.46 - 0.56	0.51 - 0.61	0.53 - 0.63	0.51 - 0.61
p value	0.53	0.43	0.56	0.04	0.01	0.03
Cut-off	> 24	> 92	≤ 35	> 65	≤ 141	≤ 7.52
Sensitivity	42.2	62.8	14.5	67	68.1	82.4
Specificity	66.3	44.3	91.5	49	48.1	34.4

Continue...

...continuation

Orotracheal intubation within > 24 hours to 7 days						
	RR	SpO_2	Flow	FiO_2	SpO_2/FiO_2	ROX index
Youden index J	0.08	0.07	0.06	0.16	0.16	0.16
Z statistic	0.61	0.78	0.57	2.04	2.56	2.1
**12 hours**						
AUROC	0.52	0.5	0.51	0.6	0.64	0.59
95%CI	0.47 - 0.57	0.45 - 0.56	0.46 - 0.56	0.55 - 0.65	0.59 - 0.69	0.54 - 0.64
p value	0.41	0.79	0.72	< 0.001	< 0.001	0.001
Cut-off	> 24	> 90	> 50	> 65	≤ 122	≤ 8.17
Sensitivity	42.2	69	60.8	70.1	65.8	89.6
Specificity	64.5	24.6	45.5	52.0	62.2	29.8
Youden index J	0.06	0.06	0.06	0.22	0.28	0.19
Z statistic	0.81	0.26	0.35	3.48	4.51	3.13
**24 hours**						
AUROC	0.62	0.55	0.53	0.64	0.63	0.66
95%CI	0.56 - 0.67	0.49 - 0.6	0.47 - 0.58	0.58 - 0.69	0.58 - 0.68	0.61 - 0.71
p value	< 0.001	0.13	0.30	< 0.001	< 0.001	< 0.001
Cut-off	> 23	≤ 93	> 55	> 62	≤ 123	≤ 7.25
Sensitivity	63.5	63.5	61.4	76	67.6	89.5
Specificity	58.8	46.5	44.7	47.9	59.9	41.7
Youden index J	0.22	0.1	0.06	0.24	0.27	0.31
Z statistic	3.66	1.49	1.02	4.62	3.9	5.51

Table 5S - Comparison of respiratory oxygenation indexes area under the receiver operating characteristics to orotracheal intubation within 24 hours, 48 hours and 7 days

	Difference between areas	95%CI	Z statistic	p value
**Orotracheal intubation within 24 hours**				
**ROX-baseline**				
ROX-2 h	0.01	-0.03 - 0.06	0.65	0.51
ROX-4 h	0.03	-0.01 - 0.09	1.33	0.18
ROX-6 h	0.04	-0.01 - 0.09	1.51	0.12
ROX-12 h	0.11	0.06 - 0.17	4.08	< 0.001
**ROX-2h**				
ROX-4 h	0.02	-0.03 - 0.07	0.77	0.43
ROX-6 h	0.02	-0.02 - 0.07	1.04	0.29
ROX-12 h	0.09	0.04 - 0.15	3.54	< 0.001
**ROX-4h**				
ROX-6 h	0.005	-0.03 - 0.05	0.23	0.81
ROX-12 h	0.07	0.02 - 0.12	3.02	0.002
**ROX-6h**				
ROX-12 h	0.07	0.02 - 0.11	3.05	0.002
**Orotracheal intubation within 48 hours**				
**ROX-baseline**				
ROX-2 h	0.01	-0.03 - 0.06	0.63	0.52
ROX-4 h	0.01	-0.04 - 0.07	0.47	0.63
ROX-6 h	0.05	0.001 - 0.11	2.02	0.04
ROX-12 h	0.11	0.05 - 0.17	3.6	< 0.001
ROX-24 h	0.18	0.11 - 0.24	5.23	< 0.001
**ROX-2h**				
ROX-4 h	0.001	-0.05 - 0.05	1.64	0.94
ROX-6 h	0.04	-0.008 - 0.09	1.64	0.10
ROX-12 h	0.09	0.03 - 0.15	3.25	0.001
ROX-24 h	0.16	0.09 - 0.22	4.91	< 0.001
**ROX-4 h**				
ROX-6 h	0.04	0.001 - 0.08	2.01	0.04
ROX-12 h	0.10	0.04 - 0.15	3.9	< 0.001
ROX-24 h	0.16	0.11 - 0.22	5.98	< 0.001
**ROX-6 h**				
ROX-12 h	0.05	0.008 - 0.1	2.3	0.02
ROX-24h	0.12	0.06 - 0.17	4.49	< 0.001
**ROX-12 h**				
ROX-24 h	0.06	0.01 - 0.11	2.59	0.009
**Orotracheal intubation within 7 days**				
**ROX-baseline**				
ROX-2 h	0.003	-0.04 - 0.04	0.14	0.88
ROX-4 h	0.004	-0.04 - 0.05	0.16	0.86
ROX-6 h	0.03	-0.01 - 0.08	1.33	0.18

Continue...

...continuation

**Orotracheal intubation within 7 days**				
ROX-12 h	0.07	0.02 - 0.13	2.7	0.006
ROX-24 h	0.14	0.08 - 0.2	4.83	< 0.001
**ROX-2 h**				
ROX-4 h	0.007	-0.03 - 0.05	0.32	0.74
ROX-6 h	0.03	-0.06 - 0.08	1.66	0.09
ROX-12 h	0.08	0.02 - 0.13	3.07	0.002
ROX-24 h	0.14	0.09 - 0.2	5.3	< 0.01
**ROX-4 h**				
ROX-6 h	0.03	-0.008 - 0.07	1.54	0.12
ROX-12 h	0.07	0.02 - 0.11	3.12	0.001
ROX-24 h	0.14	0.09 - 0.19	5.48	< 0.001
**ROX-6 h**				
ROX-12 h	0.04	-0.0005 - 0.08	1.93	0.05
ROX-24 h	0.1	0.06 - 0.15	4.69	< 0.001
**ROX-12h**				
ROX-24 h	0.06	0.02 - 0.11	2.94	0.003

AUROC - area under the receiver operating characteristic; 95%CI - 95% confidence interval; ROX - respiratory oxygenation index.

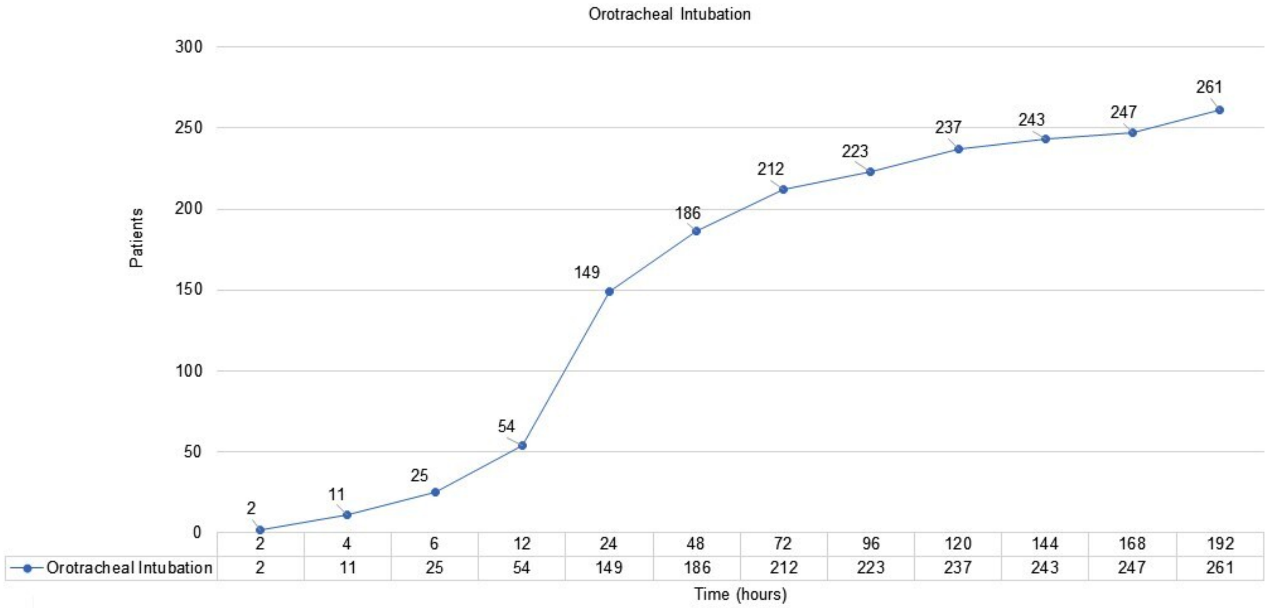

Figure 1S - Analysis of the number of intubated patients with time (hours) after starting high-flow nasal cannula.

A

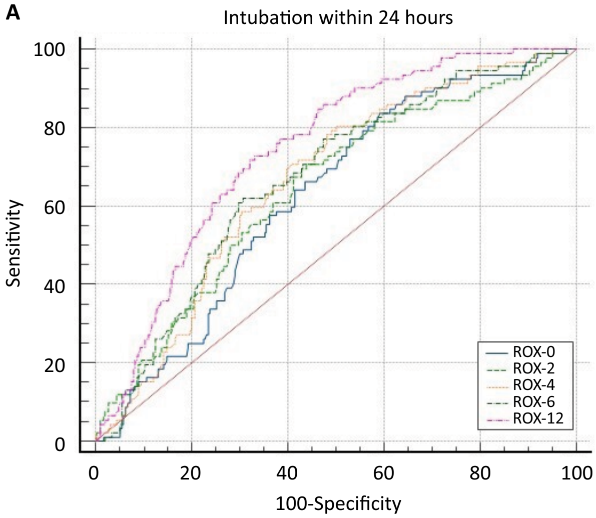

	ROX baseline	ROX-2h	ROX-4h	ROX-6h	ROX-12h
ROX baseline	**0.65 (0.58-0.69)**	0.01 (-0.03-0.06)	0.03 (-0.01-0.09)	0.04 (-0.01-0.09)	0.11 (0.06-0.17)
ROX-2h	0.51	**0.69 (0.64-0.73)**	0.02 (-0.03-0.07)	0.02 (-0.02-0.07)	0.09 (0.04-0.15)
ROX-4h	0.18	0.43	**0.69 (0.64-0.73)**	0.005 (-0.03-0.05)	0.07 (0.02-0.12)
ROX-6h	0.12	0.29	0.81	**0.70 (0.65-0.74)**	0.07 (0.02-0.12)
ROX-12h	<0.001	<0.001	0.002	0.002	**0.74 (0.69-0.78)**

B

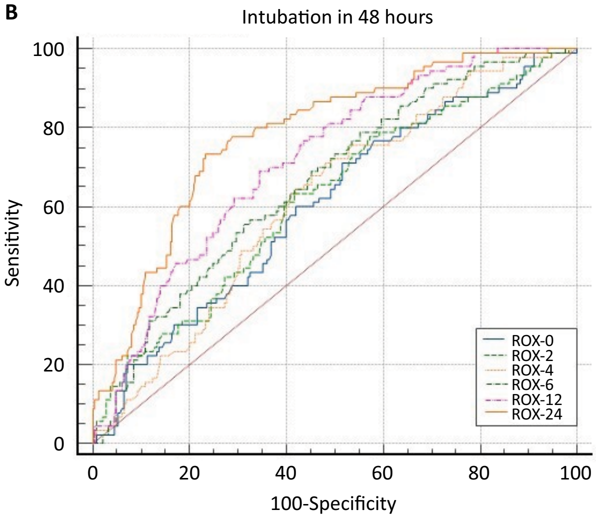

	ROX baseline	ROX-2h	ROX-4h	ROX-6h	ROX-12h	ROX-24h
ROX baseline	**0.65 (0.61-0.70)**	0.01 (-0.03-0.06)	0.01 (-0.04-0.07)	0.05 (0.001-0.11)	0.11 (0.05-0.15)	0.18 (0.11-0.24)
ROX-2h	0.52	**0.67 (0.62-0.71)**	0.001 (-0.05-0.05)	0.04 (-0.008-0.09)	0.09 (0.03-0.15)	0.16 (0.09-0.22)
ROX-4h	0.63	0.94	**0.65 (0.60-0.69)**	0.04 (0.001-0.08)	0.10 (0.04-0.15)	0.16 (0.11-0.22)
ROX-6h	0.04	0.10	0.04	**0.69 (0.64-0.73)**	0.05 (0.001-0.08)	0.12 (0.06-0.17)
ROX-12h	<0.001	0.001	<0.001	0.02	**0.73 (0.68-0.77)**	0.06 (0.01-0.11)
ROX-24h	<0.001	<0.001	<0.001	<0.001	0.009	**0.78 (0.73-0.82)**

Figure 2S - Area under the receiver operating characteristic curve and 95% confidence intervals for respiratory oxygenation index measurements at different time intervals from the start of high-flow nasal cannula therapy (baseline, 2, 4, 6, 12 and 24 hours) and its association with orotracheal intubation within: A) 24 hours; B) 48 hours.

The data in the dark blue cells on the central diagonal refers to the areas under the curves of the area under the receiver operating characteristic of the respiratory oxygenation index measured at different times since the installation of the high-flow nasal cannula. Below the area under the receiver operating characteristic data cells are the p values for comparisons between respiratory oxygenation index measurement time points. Above the area under the receiver operating characteristic information, we find the differences between the areas under the curve between the different moments.
ROX - respiratory oxygenation index.
