# Supplementary material for: The respiratory oxygenation index for identifying the risk of orotracheal intubation in COVID-19 patients receiving high-flow nasal cannula oxygen
Source: Crit Care Sci. 2024 Jun 14;36:e20240203en. doi: 10.62675/2965-2774.20240203-en (PMC11208043; doi:10.62675/2965-2774.20240203-en)
Supplement: Translation

# Translation: Índice de oxigenação respiratória para identificar risco de intubação orotraqueal em pacientes com COVID-19 que recebem oxigênio por cânula nasal de alto fluxo

**DOI:** 10.62675/2965-2774.20240203-pt

**Authors:** Aline Braz Pereira, Felipe Dal Pizzol, Viviane Cordeiro Veiga, Leandro Utino Taniguchi, Aline Finoti Misquita, Gustavo Augusto Couto Carvalho, Ligia Maria Coscrato Junqueira Silva, Michelli Marcela Dadam, Ruthy Perotto Fernandes, Israel Silva Maia, Cassio Luis Zandonai, Alexandre Biasi Cavalcanti, Marcelo Luz Pereira Romano, Glauco Adrieno Westphal

**Affiliations:** 1 Joinville SC Brasil Unidade de Terapia Intensiva, Centro Hospitalar Unimed, Joinville (SC), Brasil.; 2 Criciúma SC Brasil Programa de Pós-Graduação em Ciências da Saúde, Universidade do Extremo Sul Catarinense - Criciúma (SC), Brasil.; 3 São Paulo SP Brasil Unidade de Terapia Intensiva, BP - A Beneficência Portuguesa de São Paulo - São Paulo (SP), Brasil.; 4 São Paulo SP Brasil Unidade de Terapia Intensiva, Hospital das Clínicas, Faculdade de Medicina, Universidade de São Paulo - São Paulo (SP), Brasil.; 5 Joinville SC Brasil Unidade de Terapia Intensiva, Hospital Municipal São José - Joinville (SC), Brasil.; 6 Florianópolis SC Brasil Unidade de Terapia Intensiva, Hospital Nereu Ramos - Florianópolis (SC), Brasil.; 7 São Paulo SP Brasil Unidade de Terapia Intensiva, Hcor-Hospital do Coração, Associação Beneficente Síria - São Paulo (SP), Brasil.

**Keywords:** COVID-19, Infecções por coronavírus, Cânula, Intubação, Insuficiência respiratória, Taxa respiratória, Oxigenação

## Abstract

**Objetivo:**

Avaliar se o índice de oxigenação respiratória medido após o início da terapia de oxigênio com cânula nasal de alto fluxo pode ajudar a identificar a necessidade de intubação em pacientes com insuficiência respiratória aguda devido à COVID-19.

**Métodos:**

Este estudo retrospectivo, observacional e multicêntrico foi realizado nas unidades de terapia intensiva de seis hospitais brasileiros, de março a dezembro de 2020. O desfecho primário foi a necessidade de intubação até 7 dias após o início da cânula nasal de alto fluxo.

**Resultados:**

O estudo incluiu 444 pacientes; 261 (58,7%) foram submetidos à intubação. Uma análise da área sob a curva *receiver operating characteristic* (ASC ROC) mostrou que a capacidade de discriminar entre o sucesso e o fracasso da oxigenoterapia com cânula nasal de alto fluxo dentro de 7 dias foi maior para o índice de oxigenação respiratória medido em 24 horas (ASC ROC 0,80; IC95% 0,76 - 0,84). O intervalo médio entre o início da cânula nasal de alto fluxo e a intubação foi de 24 horas (24 - 72), e o preditor mais preciso de intubação obtido antes de 24 horas foi o índice de oxigenação respiratória medido em 12 horas (ASC ROC 0,75; IC95% 0,70 - 0,79). As curvas de Kaplan-Meier revelaram maior probabilidade de intubação em 7 dias em pacientes com índice de oxigenação respiratória ≤ 5,54 em 12 horas (razão de risco 3,07; IC95% 2,24 - 4,20) e ≤ 5,96 em 24 horas (razão de risco 5,15; IC95% 3,65 - 7,27).

**Conclusões:**

O índice de oxigenação respiratória pode ajudar na identificação precoce de pacientes com insuficiência respiratória aguda devido à COVID-19 que evoluirão para o fracasso da terapia de suporte com cânula nasal de alto fluxo e a necessidade de intubação.

## INTRODUÇÃO

A cânula nasal de alto fluxo (CNAF) é um sistema de fornecimento de oxigênio que pode ofertar até 100% de oxigênio aquecido e umidificado através da interface nasal a uma taxa de fluxo máxima de 60L/minuto (alguns dispositivos permitem fluxo máximo de 80L/minuto).^(1)^ Além de permitir o fornecimento de altas frações de oxigênio inspirado (FiO_2_), o uso da CNAF pode melhorar a eficiência ventilatória, reduzir o espaço morto e favorecer a diminuição do dióxido de carbono.^(2)^

A oxigenoterapia com CNAF tem recebido atenção como estratégia de suporte ventilatório não invasivo em pacientes com pneumonia e hipoxemia aguda grave. Essa técnica tem sido associada à melhora da ventilação alveolar e à redução do esforço respiratório.^(3-6)^ Em pacientes com doença pelo coronavírus 2019 (COVID-19), a terapia com CNAF pode reduzir a necessidade de intubação, bem como o tempo de internação na unidade de terapia intensiva (UTI), sem efeito aparente sobre a mortalidade.^(7-9)^ Embora o uso da terapia com CNAF em pacientes hipoxêmicos esteja associado a resultados positivos,^(10,11)^ a intubação tardia pode levar a desfechos desfavoráveis, incluindo aumento da mortalidade.^(12-14)^ As CNAFs têm sido amplamente utilizadas dentro e fora da UTI, e reconhecer os pacientes que se deteriorarão e precisarão de internação na UTI e ventilação mecânica (VM) é extremamente importante.^(15)^

O índice de oxigenação respiratória (índice ROX), que é definido como a razão entre a saturação periférica de oxigênio (SpO_2_) e a FiO_2_ dividida pela frequência respiratória, foi proposto como medida para identificar os pacientes que estão em maior risco de falha do suporte não invasivo com a terapia de CNAF durante a insuficiência respiratória hipoxêmica. Em pacientes com pneumonia e insuficiência respiratória hipoxêmica aguda, o índice ROX medido dentro de 12 horas após o início da terapia de CNAF é um bom preditor de maior risco de falha da CNAF.^(16,17)^ Diferentes estudos demonstraram que índice ROX ≤ 4,88 medido nas primeiras horas da terapia de CNAF tem boa capacidade discriminatória para identificar o risco de intubação em pacientes hipoxêmicos,^(18,19)^ incluindo aqueles com COVID-19.^(20-23)^ O índice ROX poderia refletir um momento específico no tempo em vez da evolução clínica do paciente, e alguns autores também sugeriram que a pontuação do índice ROX no momento da intubação estava associada a melhor sobrevida até a alta hospitalar e pode refletir a gravidade da doença respiratória.^(24)^

No entanto, poucos estudos analisaram o índice ROX em pacientes com COVID-19. A maioria deles eram estudos realizados em um único centro com amostras pequenas. Nenhum foi realizado no Brasil. As metanálises mostraram que a heterogeneidade entre os estudos era alta, e foram usados diferentes valores de corte do índice ROX. O objetivo deste estudo foi avaliar se o índice ROX medido após o início da terapia com CNAF pode ajudar a identificar a necessidade de intubação em pacientes com insuficiência respiratória aguda devido à COVID-19.

## MÉTODOS

### Desenho e cenário do estudo

Trata-se de estudo retrospectivo, observacional e multicêntrico realizado nas UTIs de seis hospitais brasileiros de março a dezembro de 2020. Os Comitês de Ética em Pesquisa de todos os centros aprovaram o protocolo do estudo (Certificado de Apresentação de Apreciação Ética: 46574321.1.1001.5362). Como este estudo foi retrospectivo, foi dispensado o consentimento informado.

### Participantes

Incluímos pacientes com mais de 18 anos de idade, internados na UTI, com insuficiência respiratória aguda devido à COVID-19 confirmada e que estavam recebendo oxigenoterapia com CNAF. A insuficiência respiratória aguda foi diagnosticada com base no julgamento clínico das equipes. A presença da COVID-19 foi confirmada por reação em cadeia da polimerase com transcrição reversa (RT-PCR), teste de antígeno ou teste sorológico (imunoglobulina M [IgM] positivo).^(25,26)^

Os critérios de exclusão foram a presença de insuficiência respiratória aguda sem confirmação laboratorial da COVID-19 e com diagnóstico alternativo mais provável ou confirmação laboratorial de outro agente etiológico; intubação orotraqueal na admissão na UTI; terapia com CNAF pós-extubação ou pós-operatória; doença em estágio terminal ou cuidados paliativos exclusivos; e prontuários incompletos em relação aos dados do desfecho primário.

### Desfechos

O desfecho primário foi a necessidade de intubação orotraqueal até 7 dias após o início da terapia com CNAF. O desfecho secundário foi a necessidade de intubação orotraqueal dentro de 48 horas após o início da terapia com CNAF. Em termos de critérios de intubação, não foi realizada padronização formal entre os centros participantes, pois não havia critérios validados para intubação orotraqueal de pacientes com COVID-19 durante o período do estudo.

### Fontes de dados e medição

As seguintes variáveis foram coletadas e registradas para análise: sexo, idade e *Simplified Acute Physiology Score* 3 (SAPS 3). Dados fisiológicos como frequência cardíaca, pressão arterial média, frequência respiratória, SpO_2_, FiO_2_ e fluxo de oxigênio foram registrados pelo menos a cada 2 horas em formulários de monitoramento de pacientes à beira do leito, conforme os procedimentos de rotina dos serviços. O índice ROX foi calculado usando a seguinte fórmula: (SpO_2_/FiO_2_)/frequência respiratória. As variáveis foram obtidas dos prontuários médicos, e o índice ROX foi calculado na seguinte ordem cronológica: início da terapia com CNAF (ROX basal) e após 2 (ROX 2 h), 6 (ROX 6 h), 12 (ROX 12 h) e 24 horas (ROX 24 h). Cada paciente foi acompanhado até a alta hospitalar ou morte. Os dados foram coletados dos prontuários médicos dos pacientes e transferidos para um formulário de relatório de caso impresso ou eletrônico. Durante e após o término da coleta de dados, os coordenadores de pesquisa do centro de coordenação do estudo mantiveram contato com os centros participantes para garantir o preenchimento correto dos dados e o uso de informações confiáveis para evitar a falta de dados e mitigar o risco de viés.

### Tamanho da amostra

O tamanho mínimo estimado da amostra para confirmar ou refutar a hipótese do estudo foi de 243 pacientes, assumindo erro do tipo 1 de 0,05, erro do tipo 2 de 0,20, área sob a curva *receiver operating characteristic* (ASC ROC) de 0,80 e hipótese nula de ASC ROC igual a 0,70, considerando taxa de intubação de 28 a 35%, com base em estudos anteriores.^(18,19,27)^

### Métodos estatísticos

Todas as análises foram realizadas com o MedCalc Statistical Software, versão 20.2 (MedCalc Software Ltd., Ostend, Bélgica) [https://www.medcalc.org; 2022]. O teste de Kolmogorov-Smirnov foi usado para avaliar a distribuição dos dados. As variáveis contínuas são relatadas como medianas e os respectivos intervalos interquartis (IIQs) e foram comparadas usando o teste U de Mann-Whitney. As variáveis categóricas são apresentadas como frequências absolutas e relativas e foram comparadas com o teste do qui-quadrado. Valor de p < 0,05 foi considerado indicador de significância estatística. No entanto, os resultados do desfecho secundário e outras análises devem ser considerados exploratórios (intervalo de confiança de 95% [IC95%] e valor de p) porque não foram ajustados para testes de hipóteses múltiplas. Verificamos a ASC ROC, para determinar o índice ROX que define o sucesso ou fracasso da oxigenoterapia com CNAF no início dessa terapia e após 2, 6, 12 e 24 horas. No caso de ASC ROC de 0,70 a 0,79, isso indica capacidade discriminatória moderada, e ASC ROC ≥ 0,80 indica discriminação excelente.^(27)^ Também foram obtidas as respectivas sensibilidade e especificidade. Após definir o melhor índice ROX na análise ASC ROC correspondente à maximização do índice de Youden, as curvas de Kaplan-Meier foram construídas para analisar o tempo até a intubação, e os grupos foram comparados usando o teste de *log-rank*. Foi realizada uma análise de sensibilidade do desfecho primário entre os pacientes que foram intubados antes e depois do intervalo médio entre o início da terapia com CNAF e a intubação para reduzir o efeito do viés de seleção. Os pacientes com registros incompletos do desfecho primário foram excluídos do estudo.

## RESULTADOS

### Participantes

Internaram-se 489 pacientes em UTIs por causa de insuficiência respiratória devido à COVID-19 e receberam terapia com CNAF. Desses, 444 atenderam aos critérios de seleção e foram incluídos no estudo (Figura 1); 261 (58,7%) evoluíram para intubação. Os pacientes intubados eram mais jovens (59 [49 - 73] *versus* 65 [54 - 77] anos; p = 0,001), mais graves (SAPS 3: 44 [37 - 52] *versus* 42 [36 - 47]; p < 0,001) e necessitavam mais frequentemente de ventilação não invasiva por máscara facial (69,3% *versus* 58,4%; p = 0,01). Os grupos eram semelhantes em termos de comorbidades, pontuação do Índice de Comorbidade de Charlson (ICC), posicionamento em decúbito ventral acordado e tipos de dispositivos de fornecimento de oxigênio usados antes da terapia com CNAF (Tabela 1).

**Figura 1:** Seleção dos pacientes.

CNAF - cânula nasal de alto fluxo; VM - ventilação mecânica; UTI - unidade de terapia intensiva.

**Tabela 1:** Dados clínicos de pacientes internados em unidades de terapia intensiva que receberam oxigenoterapia com cânula nasal de alto fluxo

		Total (n = 444)	Intubados (n = 261)	Não intubados (n = 183)	Valor de p
Idade (anos)		61 (52 - 75)	59 (49 - 73)	65 (54 - 77)	0,001
Sexo masculino		292 (65,7)	176 (67,4)	116 (63,3)	0,38
SAPS 3		43 (37 - 50)	44 (37 - 52)	42 (36 - 47)	< 0,001
Comorbidades					
	Cardiovasculares	89 (20)	55 (21)	34 (18,5)	0,51
	Respiratórias	24 (5,4)	16 (6,1)	8 (4,3)	0,41
	Neurológicas	7 (1,5)	5 (1,9)	2 (1)	0,49
	Gastrenterológicas	9 (2)	6 (2,2)	3 (1,6)	0,62
	Renais/metabólicas	87 (19,5)	56 (21,4)	31 (16,6)	0,23
	Neoplasias	29 (6,5)	21 (8)	8 (4,3)	0,12
	Imunológicas	7 (1,5)	5 (1,9)	2 (1)	0,49
Charlson		3 (2 - 4)	3 (1 - 4)	3 (2 - 4)	0,45
Dispositivo antes da CNAF					
	Cateter nasal	74 (16,6)	40 (15,3)	34 (18,5)	0,36
	Máscara facial tipo tenda/macronebulizador	3 (0,6)	2 (0,7)	1 (0,5)	0,78
	Máscara com reservatório	357 (80,4)	215 (82,3)	142 (77,5)	0,21
	Máscara de Venturi	5 (1,1)	1 (0,3)	4 (2,1)	0,07
Posicionamento em decúbito ventral acordado		251 (56,5)	145 (55,5)	106 (57,9)	0,62
Ventilação não invasiva		288 (64,8)	181 (69,3)	107 (58,4)	0,01

SAPS 3 - *Simplified Acute Physiology Score*; CNAF - cânula nasal de alto fluxo. Valor de p: comparação entre pacientes intubados e não intubados. Resultados apresentados como medianas (intervalos interquartis) ou n (%).

Durante o seguimento, os pacientes intubados apresentaram maior tempo de internação na UTI (p < 0,001) e no hospital (p < 0,001) e maior mortalidade na UTI e no hospital (p < 0,001) (Tabela 2). Todas as mortes ocorreram após a intubação. Entre os pacientes que necessitaram de suporte ventilatório invasivo, 245 (94,5%) foram intubados em até 7 dias, e 184 (71,0%) foram intubados dentro de 48 horas após o início da terapia com CNAF (Figura 1S - Material Suplementar). A duração da VM foi de 10 dias (5 - 21) (Tabela 1S - Material Suplementar).

**Tabela 2:** Dados de seguimento de pacientes internados em unidades de terapia intensiva que receberam oxigenoterapia com cânula nasal de alto fluxo

		Total (n = 444)	Intubados (n = 261)	Não intubados (n = 183)	Valor de p
Tempo de internação na UTI (dias)		11 (7 - 21)	18 (11-28)	7 (5 - 10)	< 0,001
Tempo de internação hospitalar (dias)		19 (12 - 31)	25 (15 - 40)	14 (10 - 21)	< 0,001
Morte hospitalar		123 (27,7)	123 (47,1)	0 (0)	< 0,001
Causa da morte					
	Hipoxemia refratária	60 (48,7)	60 (48,7)	0 (0)	-
	Choque refratário	38 (30,8)	38 (30,8)	0 (0)	-
	Disfunção de múltiplos órgãos	21 (17)	21 (17)	0 (0)	-
	Outras	4 (3,2)	4 (3,2)	0 (0)	-

UTI - unidade de terapia intensiva. Valor de p: comparação entre pacientes intubados e não intubados. Resultados apresentados como medianas (intervalos interquartis) ou n (%).

### Parâmetros respiratórios

Ao comparar os sinais vitais basais, a SpO_2_, os parâmetros ajustados durante a terapia com CNAF e o índice ROX entre pacientes intubados e não intubados em diferentes durações da terapia com CNAF, observamos que a frequência respiratória foi semelhante entre os grupos basais até a sexta hora, mas foi maior nas primeiras 12 horas (24 [21 - 27] *versus* 22 [19 - 25]; p = 0,002) e 24 horas (25 [22 - 27] *versus* 22 [19 - 24]; p < 0,001) após o início da terapia com CNAF. Os pacientes intubados também apresentaram fluxo e FiO_2_ significativamente mais altos e índice ROX mais baixo em todos os momentos, enquanto a SpO_2_ foi mais baixa após a sexta hora (Tabela 2S - Material suplementar).

### Precisão dos diferentes parâmetros

**Desfecho primário:** uma análise das ASC ROCs revelou maior capacidade de discriminar entre o sucesso e o fracasso da terapia com CNAF dentro de 7 dias para o índice ROX 24 horas (ASC ROC 0,80; IC95% 0,76 - 0,84), seguido por SpO_2_/FiO_2_ em 24 horas (ASC ROC 0,76; IC95% 0,71 - 0,80). O intervalo médio entre o início da terapia com CNAF e a intubação foi de 24 horas (24 - 72), e o preditor mais preciso de intubação medido antes de 24 horas foi o índice ROX 12 h (ASC ROC 0,75; IC95% 0,70 - 0,79), seguido pelo índice ROX 6 h (ASC ROC 0,71; IC95% 0,67 - 0,76). Utilizando-se o melhor valor de corte de cada um desses parâmetros, o índice ROX 24 h ≤ 5,96 apresentou sensibilidade de 80,6% e especificidade de 68,9% para prever a necessidade de intubação em 7 dias em comparação com SpO_2_/FiO_2_ ≤ 129 em 24 horas (sensibilidade: 71,0%; especificidade: 70,2%), índice ROX 12 h ≤ 5,54 (sensibilidade: 67,3%; especificidade: 72,8%) e índice ROX 6 h ≤ 6,08 (sensibilidade: 69,4%; especificidade: 65,9). Quando o índice ROX 12 h ≤ 4,88 (relatado como o valor de corte do índice ROX em pacientes sem COVID-19)^(19,20)^ foi usado arbitrariamente, a sensibilidade foi de 57% (IC95% 47 - 72) e a especificidade, 78% (IC95% 72 - 84; índice de Youden J = 0,40; estatística Z = 10,1). As tabelas 3A e 3S (Material suplementar) mostram a sensibilidade, a especificidade e os valores preditivos positivos e negativos para os diferentes pontos de corte de cada parâmetro.

**Desfecho secundário:** em geral, uma análise da intubação em 48 horas revelou ASC ROCs ligeiramente mais baixas do que as observadas na análise de 7 dias (Tabela 3 e Tabela 3S - Material suplementar).

**Tabela 3:** Análise da área sob a curva *receiver operating characteristic* e valores de corte do índice de oxigenação respiratória para identificar o sucesso ou a falha da terapia com cânula nasal de alto fluxo e a necessidade de intubação orotraqueal em 7 dias e 48 horas

Intubação orotraqueal em 7 dias								
	ASC ROC	IC95%	Valor de p	Corte	Sensibilidade	Especificidade	Índice de Youden J	Estatística Z
ROX basal	0,69	0,65 - 0,74	< 0,001	≤ 5,35	68,1	64,8	0,32	7,8
ROX 2 h	0,69	0,64 - 0,73	< 0,001	≤ 5,57	67	67,1	0,34	7,5
ROX 4 h	0,68	0,64 - 0,73	< 0,001	≤ 5,82	67,8	66,6	0,34	7,2
ROX 6 h	0,71	0,67 - 0,76	< 0,001	≤ 6,08	69,4	65,9	0,35	8,5
ROX 12 h	0,75	0,70 - 0,79	< 0,001	≤ 5,54	67,3	72,8	0,4	10,1
ROX 24 h	0,8	0,76 - 0,84	< 0,001	≤ 5,96	80,6	68,9	0,49	13,3
**Intubação orotraqueal em 48 horas**								
	**ASC ROC**	**IC95%**	**Valor de p**	**Corte**	**Sensibilidade**	**Especificidade**	**Índice de Youden J**	**Estatística Z**
ROX basal	0,65	0,61 - 0,7	< 0,001	≤ 5,16	66,1	59,3	0,25	5,9
ROX 2 h	0,67	0,62 - 0,71	< 0,001	≤ 5,57	67,9	59,6	0,27	6,5
ROX 4 h	0,65	0,60 -0,69	< 0,001	≤ 5,82	68,4	59,1	0,27	5,7
ROX 6 h	0,69	0,64 - 0,73	< 0,001	≤ 5,17	60,9	71,2	0,32	7,3
ROX 12 h	0,73	0,68 - 0,77	< 0,001	≤ 5,54	71,5	65,4	0,37	8,9
ROX 24 h	0,78	0,73 - 0,82	< 0,001	≤ 4,84	72,5	76,5	0,49	10,2

ASC ROC - área sob a curva *receiver operating characteristic*; IC95% - intervalo de confiança de 95%; ROX - índice de oxigenação respiratória.

### Análise de sensibilidade

Com relação aos pacientes que foram intubados em até 24 horas, o índice ROX 12 h teve ASC ROC de 0,74 (IC95% 0,69 - 0,78). Para índice ROX 12 h ≤ 5,26, a sensibilidade foi de 73,1% e a especificidade foi de 66,7%. Observou-se redução importante na precisão das variáveis analisadas na população intubada após 24 horas de oxigenoterapia com CNAF (Tabela 4S - Material suplementar).

### Comparação das curvas ROC

Na comparação entre as ASC ROCs dos índices ROX obtidos nos diferentes momentos (início da terapia e 2, 4, 6, 12 e 24 horas), foram observadas diferenças significativas a favor das medições realizadas 12 e 24 horas após o início da terapia com CNAF em comparação com as medições coletadas por até 6 horas para os desfechos primário e secundário (Figura 2 e Figura 2S e Tabela 5S - Material Suplementar). Para o subgrupo de pacientes incluídos na análise de sensibilidade, as medições de índice ROX 12 h diferiram daquelas realizadas até 6 horas após o início da terapia com CNAF (Figura 2S - Material suplementar).

**Figura 2:** Área sob a curva *receiver operating characteristic* e intervalos de confiança de 95% para medições do índice de oxigenação respiratória em diferentes intervalos de tempo do início da terapia com cânula nasal de alto fluxo (início da terapia, 2, 4, 6, 12 e 24 horas) e sua associação com a intubação orotraqueal em 7 dias.

Os dados das células azul-escuras na diagonal central referem-se às áreas sob as curvas *receiver operating characteristic* do índice de oxigenação respiratória medido em diferentes momentos após a instalação da cânula nasal de alto fluxo. Os valores de p para comparações entre os pontos de tempo de medição do índice de oxigenação respiratória são mostrados abaixo da área sob a curva *receiver operating characteristic*. As diferenças entre as áreas sob as curvas em diferentes pontos de tempo são mostradas acima da área sob a curva *receiver operating characteristic*.

ROX - índice de oxigenação respiratória.

### Curvas de Kaplan-Meier

As curvas de Kaplan-Meier mostraram maior probabilidade de intubação orotraqueal em até 7 dias entre os pacientes com índice ROX 12 hs ≤ 5,54 (razão de risco 3,07; IC95% 2,24 - 4,20) e índice ROX 24 h ≤ 5,96 (razão de risco 5,15; IC95% 3,65 - 7,27) (Figura 3).

**Figura 3:** Curvas de Kaplan-Meier.

(A) Probabilidade de intubação orotraqueal no prazo de 7 dias em pacientes com índice de oxigenação respiratória 12 horas > 5,54 *versus* pacientes com índice de oxigenação respiratória 12 horas ≤ 5,54 e (B) probabilidade de intubação orotraqueal no prazo de 7 dias em pacientes com índice de oxigenação respiratória 24 horas > 5,96 *versus* pacientes com índice de oxigenação respiratória 24 horas ≤ 5,96.

ROX - índice de oxigenação respiratória; IC95% - intervalo de confiança de 95%.

## DISCUSSÃO

Neste estudo de coorte retrospectivo, observamos que o índice ROX foi um bom preditor para identificar a necessidade de intubação em pacientes com COVID-19 na UTI que receberam terapia com CNAF. Mais precisamente, índice ROX ≤ 5,96 após 24 horas de terapia de suporte com CNAF foi mais preciso para identificar o risco de intubação em 7 dias. Embora o índice ROX 12 h ≤ 5,54 não tenha indicado a necessidade de intubação com a mesma precisão, sua capacidade discriminatória foi moderada, semelhante aos achados de outros autores.^(20-23)^

Durante a pandemia da COVID-19, a terapia com CNAF foi amplamente utilizada após a superação de dúvidas e receios em relação à segurança da equipe de saúde,^(28,29)^ sendo considerada uma alternativa muito interessante ao suporte ventilatório não invasivo em pacientes hipoxêmicos com síndrome respiratória aguda grave devido à COVID-19.^(30-37)^ A oxigenoterapia com CNAF reduziu significativamente a necessidade de VM e o tempo de recuperação clínica em comparação com a oxigenoterapia convencional, sem impacto na mortalidade ou no tempo de internação na UTI.^(7-9,38)^

A identificação precoce da necessidade de intubação em pacientes que recebem suporte ventilatório não invasivo está associada ao prognóstico do paciente.^(12)^ No entanto, essa identificação é desafiadora devido à imprecisão do uso individual de parâmetros clínicos comuns, como frequência respiratória, concentração de oxigênio e SpO_2_.^(18,19)^ Embora as evidências indiquem que a CNAF pode reduzir o esforço inspiratório de pacientes com insuficiência respiratória aguda devido à COVID-19,^(39)^ persistem incertezas quanto ao momento ideal em que a VM invasiva deve ser iniciada, bem como quanto aos riscos relativos de lesão pulmonar autoinfligida pelo paciente *versus* lesão pulmonar induzida pelo ventilador.^(40-44)^ Sabendo do valor preditivo do índice ROX no cenário pré-pandêmico, alguns autores avaliaram o potencial desse parâmetro no monitoramento de pacientes com COVID-19 para identificar a falha da terapia com CNAF ou ventilação não invasiva por máscara facial e encontraram boa precisão desse índice na previsão da necessidade de intubação.^(18-22)^ Como a CNAF foi amplamente utilizada durante a pandemia de COVID-19 na UTI e fora da UTI, o índice ROX tornou-se uma ferramenta importante para identificar os pacientes que podem piorar e necessitar de internação na UTI e ventilação mecânica.^(15)^

Com o objetivo de avaliar o desempenho preditivo do índice ROX para o sucesso do desmame da terapia com CNAF em pacientes com pneumonia e insuficiência respiratória hipoxêmica aguda, uma revisão sistemática e metanálise de 13 estudos observacionais envolvendo 1.751 pacientes revelou que o índice ROX, medido dentro de 12 horas após o início da terapia com CNAF, apresentou bom desempenho na previsão do sucesso do desmame da terapia com CNAF, com valores de corte médios e medianos do índice ROX de 4,8 (IC95% 4,2 - 5,4) e 5,3 (IC95% 4,2 - 5,5), respectivamente.^(16)^ Outros autores relataram resultados semelhantes.^(17)^ Ao analisar a precisão do índice ROX em pacientes com insuficiência respiratória aguda devido à COVID-19, outra revisão sistemática e metanálise de oito estudos com 1.301 pacientes revelou bom poder discriminatório do índice ROX na identificação do fracasso da terapia com CNAF (ASC ROC sumária 0,81; IC95% 0,77 - 0,84).^(23)^

Observamos precisão aceitável do índice ROX 12 h na discriminação de pacientes com insuficiência respiratória devido à COVID-19 que podem progredir para falha na terapia com CNAF (ASC ROC = 0,75), o que foi apoiado pela análise de sensibilidade. O intervalo médio entre o início da terapia com CNAF e a intubação foi de 24 horas, indicando que metade dos pacientes foi submetida à intubação orotraqueal dentro de 24 horas. Portanto, o índice ROX 24 h foi obtido imediatamente antes da intubação ou após a intubação em metade dos pacientes. Devemos enfatizar que o índice ROX foi determinado durante a terapia com CNAF, e os pacientes que foram intubados após 12 horas de terapia com CNAF não foram considerados na análise do índice ROX 24 h. Roca et al.^(18,19)^ relataram resultados muito semelhantes em relação à capacidade preditiva do índice ROX medido 12 horas após o início da terapia com CNAF em pacientes com insuficiência respiratória hipoxêmica devido à pneumonia comunitária. Além disso, esses autores encontraram maior precisão do índice ROX na previsão de intubação devido à falha da terapia com CNAF em comparação com outras variáveis comumente usadas, como a frequência respiratória, o fluxo de oxigênio e a SpO_2_, como também observado no presente estudo.

Nesse contexto, embora o índice ROX 24 h seja mais preciso (ASC ROC = 0,80) do que os parâmetros comumente usados, a identificação da necessidade de intubação apenas 24 horas após o início da terapia com CNAF parece ser tardia e pode afetar negativamente o prognóstico do paciente.^(12)^ Constatamos que a taxa de falhas e a necessidade de intubação ainda eram significativas dentro de 7 dias após o início da terapia com CNAF, com 75 (28,7%) pacientes intubados após 48 horas e até 7 dias (Figura 1S - Material Suplementar). Esses achados sugerem que o índice ROX pode ser usado para identificar pacientes com doença respiratória mais grave, que podem ter desfecho desfavorável e merecem maior vigilância e monitoramento em uma UTI. Entretanto, o índice ROX pode refletir um momento específico no tempo em vez da evolução clínica do paciente, e esses parâmetros podem facilmente variar ao longo do dia ou em diferentes situações clínicas (febre, mobilização, fadiga, dor, acidose e hipotensão). Esse resultado sugere que outros parâmetros, como deterioração neurológica, trabalho de respiração, alterações do estado mental, agitação, sonolência e estupor, não devem ser ignorados. Com relação à validade externa de nossos achados, o banco de dados desse estudo multicêntrico incluiu pacientes adultos graves de vários estados brasileiros que foram tratados em diferentes ambientes. Além disso, todos os pacientes tinham diagnóstico confirmado de COVID-19 e foram submetidos à oxigenoterapia com CNAF com critérios semelhantes para insuficiência respiratória aguda.

Nosso estudo tem várias limitações. Embora seja um estudo multicêntrico que envolveu instituições de diferentes locais e com diferentes características, bem como com um histórico de participação em estudos multicêntricos, ele tem todas as limitações inerentes ao seu desenho retrospectivo. A escolha do tempo com a melhor ASC ROC e do valor de corte do índice ROX com a melhor sensibilidade foram procedimentos *post hoc* e podem refletir erros aleatórios; esses procedimentos não foram validados em amostras independentes. Os centros participantes não usaram um protocolo único com critérios preestabelecidos para o uso da terapia com CNAF; portanto, podemos ter excluído inadvertidamente pacientes que atendiam aos critérios para o uso dessa terapia ou até mesmo incluído outros que não atendiam aos critérios. Da mesma forma, os critérios para definir a falha terapêutica e a indicação de intubação não foram padronizados anteriormente; no entanto, os centros participantes não careciam de recursos que pudessem atrasar a intubação. Foram utilizadas diferentes marcas de dispositivos de CNAF com diferentes características para a oxigenoterapia. Em alguns dos centros participantes, a terapia com CNAF começou a ser usada durante a pandemia, o que indica um curto período de familiarização com o método. Alguns dados não foram registrados, incluindo o intervalo entre o diagnóstico de insuficiência respiratória e o início da terapia com CNAF; além disso, a duração da ventilação não invasiva, que foi usada antes e durante a terapia com CNAF, não foi registrada ou padronizada.

## CONCLUSÃO

Nossos resultados sugerem que o índice de oxigenação respiratória pode ajudar a identificar os pacientes que evoluirão para a falha da terapia de suporte com cânula nasal de alto fluxo. Esse índice é mais preciso do que os parâmetros comumente usados, como a frequência respiratória ou a saturação periférica de oxigênio. Tais achados são importantes para auxiliar os profissionais de cuidados intensivos e de emergência na identificação precoce desses pacientes e evitar atrasos na intubação.
